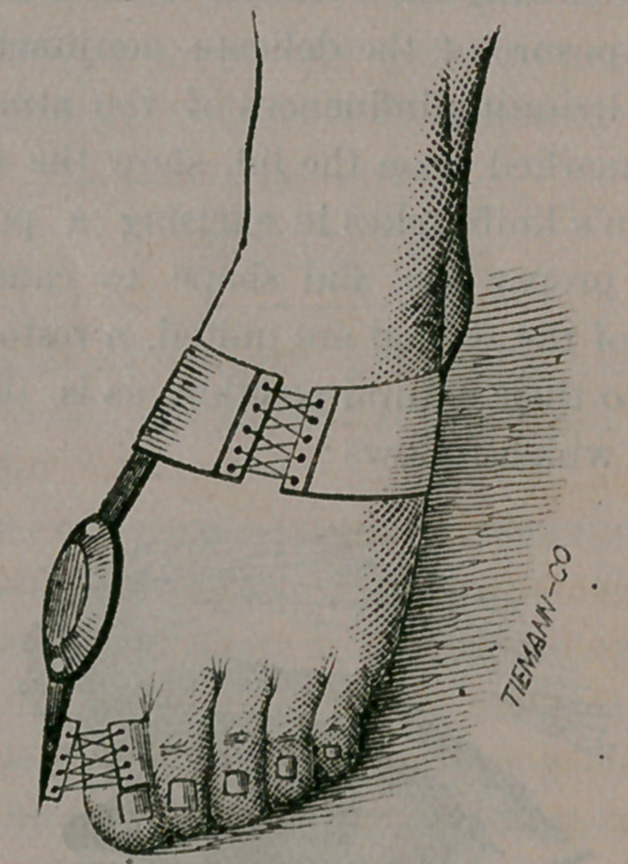# Deformities of the Feet

**Published:** 1875-04

**Authors:** 


### DEFORMITIES OF THE FEET.

In recent articles we have exhibited the various deformities of the feet, known as club-foot, and have explained the modes of cure as accomplished by the surgeon's knife and suitable apparatus. The object of the present article is to exhibit an ingenious shoe, invented by Dr. Sayre, of New York, for correcting the various forms of club-foot that depend upon paralysis of the muscles of the foot, or for the cure of the same difficulties when caused by contraction of the muscles, in young subjects. We often encounter parents who are loth to subject their children to surgical operations for rectifying difficulties of this nature, that would not hesitate a moment in applying apparatus, even should greater time be required, or more pain produced in accomplishing the cure. People have great dread, for some unaccountable reason, of the surgeon's knife, and in many instances would prefer ten times the torture accomplished by any other means. For the benefit of such we give an illustration of a very ingenious shoe, intended to overcome deformities of the feet, without cutting.

The sole, D, is a steel plate, connected with the heel, E, by a ball-and-socket joint, so that the foot can be turned to the right or left. Hooks are fastened at various points upon this metallic sole, for the attachment of artificial muscles, made of rubber, as at M, M, M. These muscles are arranged by the surgeon, to pull in any direction desired, so as to overcome contraction or paralysis of the natural muscles of the foot. Their tension is regulated by lengthening or shortening the chains seen in the cut. The apparatus is fastened to the limb by means of the two stout steel bars marked G, which are attached to the sole of the shoe, having a joint at K, allowing movements of the ankle, and fastened to the leg by means of a padded band at H. The elastic band N, is intended to keep the heel well down in the shoe.

This apparatus is adapted to almost all deformities of the feet, by arranging the artificial muscles so as to draw the foot in a direction opposite to that in which it is inclined to rest, abnormally.—Of course, a surgeon accustomed to the fitting of such appliances, is necessary in adjusting the shoe, else may more harm than good ensue from its use.

Here we picture a simple apparatus, intended to correct inturning of the toes in walking, or what is usually termed "walking pigeon-toed."

It is so simple in construction that any parent can improvise one after glancing at our engraving. Fasten two pieces of hoop-iron by means of long malleable nails, between the layers of leather of the sole and heel of each shoe. In the projecting portions have holes drilled, sufficiently large to admit the bent ends of a stout wire or rod, at the toes, of sufficient length to turn them out naturally. At the heels fasten a short chain, in a similar manner, of sufficient length to draw the heels as near together as will seem natural, and your apparatus is complete. Allow the child to run about in the house, or upon a smooth yard, for several weeks, and you will likely cure the tendency to "toeing-in." The only difficulty with this arrangement is that it is not calculated to be used in stair climbing or walking over uneven ground. We desire simply to show how such implements are constructed and used, hoping to so familiarize the people with their appearance and uses as to induce their adoption when necessity requires. One of the avowed objects of the BISTOURY is to render the public more intelligent upon medical and surgical matters, to the end that all may act understandingly both in the employment of surgeons, and of such devices as are here portrayed.

## Figures and Tables

**Figure f1:**
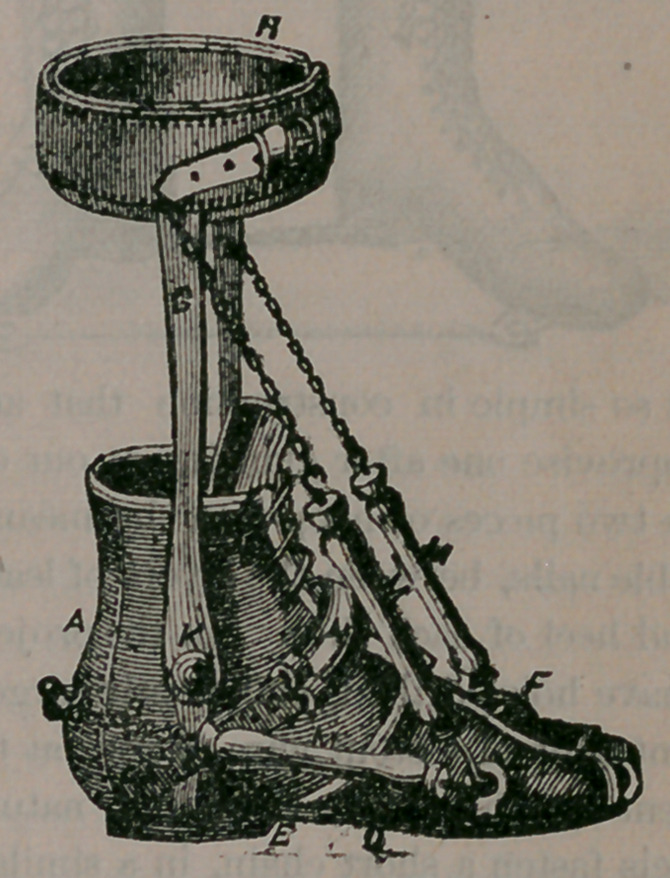


**Figure f2:**